# Microglial autophagy in cerebrovascular diseases

**DOI:** 10.3389/fnagi.2022.1023679

**Published:** 2022-10-06

**Authors:** Man Chen, Hang Zhang, Yun-Hui Chu, Yue Tang, Xiao-Wei Pang, Chuan Qin, Dai-Shi Tian

**Affiliations:** Department of Neurology, Tongji Hospital, Tongji Medical College, Huazhong University of Science and Technology, Wuhan, China

**Keywords:** microglia, autophagy, cerebrovascular diseases, inflammation, metabolism

## Abstract

Microglia are considered core regulators for monitoring homeostasis in the brain and primary responders to central nervous system (CNS) injuries. Autophagy affects the innate immune functions of microglia. Recently some evidence suggests that microglial autophagy is closely associated with brain function in both ischemic stroke and hemorrhagic stroke. Herein, we will discuss the interaction between autophagy and other biological processes in microglia under physiological and pathological conditions and highlight the interaction between microglial metabolism and autophagy. In the end, we focus on the effect of microglial autophagy in cerebrovascular diseases.

## Introduction

Microglia originate from yolk-sac progenitors and reach the brain early in the embryo to participate in the development of the CNS, such as synaptic pruning, neurogenesis, and remodeling of neural circuits (Ginhoux et al., 2010; Sierra et al., 2010; Paolicelli et al., 2011; Schafer et al., 2012). As the immune-competent cells in the brain, microglia continuously remove dead cells, cellular debris, and toxic substance (Diaz-Aparicio et al., 2020; Andoh and Koyama, 2021; Ding et al., 2021). Therefore, microglia are considered to be an important component of the development, homeostasis, and diseases of CNS. Parenchymal injuries promote the change of microglia from a steady state to a reactive state (Li and Barres, 2018). Microglial activation derives from alterations in their transcriptome, resulting in morphological and functional changes depending on the molecular characteristics of microglia (Ladeby et al., 2005; Zrzavy et al., 2017; Benmamar-Badel et al., 2020; Deng et al., 2020; West et al., 2022). Different molecular features of activated microglia have generated different terms, such as disease-associated microglia (DAM), microglial neurodegenerative phenotype (MGnD), and activation-responsive microglia (ARM) (Keren-Shaul et al., 2017; Krasemann et al., 2017; Anderson et al., 2019; Benmamar-Badel et al., 2020).

Autophagy is widespread in all mammalian cells as a highly conserved biological mechanism (Yang and Klionsky, 2010; Xiang et al., 2019). Autophagy was first thought to be used to degrade some macromolecules such as protein aggregates and damaged organelles and to counteract harsh conditions such as nutrient deprivation and genotoxic stress to promote cell survival (He and Klionsky, 2009; Su et al., 2016; Levine and Kroemer, 2019). Three different forms of autophagy have been found to exist in mammalian cells including microautophagy, chaperone-mediated autophagy (CMA), and macroautophagy (Klionsky, 2005). Although differing morphologically, they all ultimately transport cargo to lysosomes for degradation and recovery (Yang and Klionsky, 2010; Levine et al., 2011). Autophagy is a lysosomal degradation pathway that provides metabolic substrates and energy by recycling large molecules (Lum et al., 2005). Our review will discuss macroautophagy in microglia and replace it with “autophagy” below since current studies on microglial autophagy have focused on macroautophagy. Autophagy in microglia is essential for cellular homeostasis and energy balance (Julg et al., 2020; Li G. et al., 2021). In recent years, the phenomenon of microglial autophagy has been observed in various CNS diseases including neurodegenerative diseases, traumatic brain injury (TBI), infectious diseases, and cerebrovascular diseases (Jin et al., 2017; Qin et al., 2018; Guo and Buch, 2019; Pomilio et al., 2020). Meanwhile, numerous studies have shown that the regulation of microglial autophagy can play an important role in the progression and prognosis of these diseases (Su et al., 2016; Plaza-Zabala et al., 2017; Li et al., 2020; Mo et al., 2020; Estfanous et al., 2021; Zeng et al., 2022). Some studies suggest that microglial autophagy belongs to a part of its immune function (Lu et al., 2021). The regulation and effect of microglial autophagy have attracted increasing attention. The theme of the present review is to discuss the interaction between microglial autophagy and other biological processes, highlight the role of microglial autophagy in cerebrovascular diseases, and explore the feasibility of microglial autophagy as a therapeutic target for cerebrovascular diseases.

## Interaction between microglial autophagy and other biological processes

**FIGURE 1:**
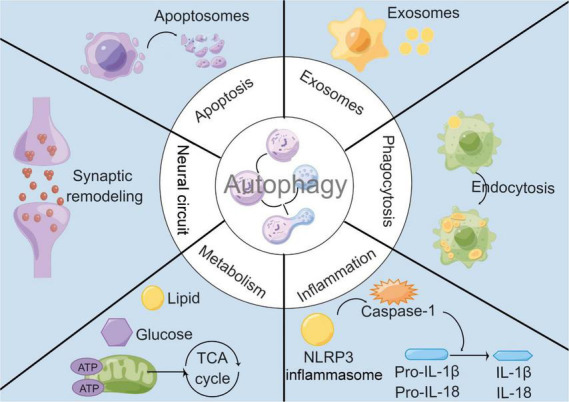
Interactions between microglial autophagy and other biological processes. Under both physiological and pathological conditions, microglial autophagy is associated with other biological processes including apoptosis, exosomes, inflammation, phagocytosis, metabolism, and neural circuit.

Although autophagy has been found to regulate innate and adaptive immune responses of the peripheral immune system, such as inflammatory responses, phagocytosis, and antigen presentation (Plaza-Zabala et al., 2017), autophagy is not an independent biological process in microglia. It is tightly linked to other biological processes through complex molecular networks to achieve an organic dynamic balance (Figure 1). Here, we will discuss the interaction of microglial autophagy with other biological processes under physiological and pathological conditions, particularly in cerebrovascular diseases.

### Autophagy and apoptosis

Apoptosis is required for normal CNS growth and development. However, excessive or insufficient apoptosis contributes to the development of various CNS diseases especially neurodegenerative diseases (Rubinsztein, 2006; Ghavami et al., 2014). As fundamental physiological processes to maintain homeostasis, the balance between autophagy and apoptosis is critical for cell fate. Normally, autophagy can protect cells from apoptosis in the CNS since autophagy degrades dysfunctional or damaged organelles, particularly mitochondria, thereby reducing the release of the priming signals for apoptosis such as cytochrome-c (Viscomi et al., 2012). For example, neuron-specific knockout of autophagy-related proteins Atg5 or Atg7 has been found to result in neuronal apoptosis *in vivo* (Maiuri et al., 2007), while enhanced autophagy can delay the neuronal apoptotic progression (Viscomi et al., 2012). However, abnormal or disordered autophagy promotes cell apoptosis. Studies revealed that ischemia/reperfusion (I/R) injury significantly activated ER stress-dependent autophagy thereby inducing neuronal apoptosis *in vivo* (Feng et al., 2017). Besides, inhibition of oxygen–glucose deprivation/reoxygenation (OGD/R)-induced autophagy prevented neuronal apoptosis in primary microglia *in vitro* (Zeng et al., 2022).

The interaction between autophagy and apoptosis in microglia in cerebrovascular diseases is controversial. Huang et al. found that primary microglia produced large amounts of reactive oxygen species (ROS) due to oxidative stress in OGD/R injury. Additionally, they revealed that enhanced autophagy could reduce the level of ROS and reduce oxidative stress-related apoptosis (Huang T. et al., 2021), whereas Chen et al. (2016) found that inhibition of autophagy could reduce microglial apoptosis/death in OGD/R injury in BV2 microglia. Yang et al. (2014c) also demonstrated that autophagy promoted hypoxia-induced cell death in primary microglia. It should be noted that the current boundary between autophagic cell death and apoptosis is not clear. Therefore, the interaction and detailed mechanism between apoptosis and autophagy in microglia need more studies to elucidate. The balance between autophagy and apoptosis determines the outcome of microglia (Zhang et al., 2016). Adjusting the microglial autophagy to keep it at an appropriate level is conducive for microglia to play a neuroprotective role in the progression of cerebrovascular diseases.

### Autophagy and exosomes

Exosomes are nanoscale extracellular vesicles produced by multivesicular bodies (MVB) that fuse with the cell membrane (Xu et al., 2018). Exosomes were first recognized as a way for cells to excrete waste products (Zhang et al., 2020). Current studies have found that exosomes can serve as a new pathway for information exchange between cells. Exosomes can participate in intercellular communication by receptor-ligand interactions, internalized by endocytosis and/or phagocytosis, or even fused with the cell membrane of the target cell (Vlassov et al., 2012). The autophagic process may be one of the downstream effects of exosomes and exosome secretion is regulated by the autophagy system (Fader and Colombo, 2006; Fader et al., 2008; Ojha et al., 2017). Microglia, as innate immune cells in the CNS, can release exosomes thereby regulating the autophagic function of other cells such as neurons (Li P. et al., 2022). And the contents and release of microglial exosomes may be affected by microglial autophagy (Olanrewaju and Hakami, 2020; Zang et al., 2020). Meanwhile, exosomes derived from other cells can act on microglia thereby affecting the autophagic function of microglia. For instance, exosomes containing α-Syn can spread mutually between neurons and glial cells during the pathogenesis of Parkinson’s disease (PD). When the exosomes are gradually transported to microglia, aggregated α-Syn impairs mitophagy and promotes secretion of proinflammatory cytokines, which leads to deterioration of neuroinflammation (Zhao and Yang, 2021). Zhou et al. (2019) also have found that exosomes released by neuronal cells transfected with α-Syn contain overexpressed mir-19a-3p, which act on microglia and inhibit autophagy *via* AKT/mTOR signaling pathway.

Cross-acting mechanisms of microglial autophagy and exosomes play important roles in cerebrovascular diseases. Recent studies have shown that exosome-mediated delivery of miR-30d-5p inhibits microglial autophagy, promotes the transition of microglia to an anti-inflammatory phenotype, suppresses the inflammatory response after ischemia, and significantly reduces the area of infarct brain injury (Jiang et al., 2018). Exosomes derived from mesenchymal stem cells were demonstrated to activate mitophagy in microglia, thereby preventing the pyroptosis of OGD/R-exposed BV2 microglia *in vitro* (Hu et al., 2021). It should be noted that Zang et al. (2020) revealed Vinpocetine could change the exosome contents of BV2 microglia by enhancing autophagic flux in OGD model, thereby promoting neuronal survival under cerebral ischemic conditions. Based on these, we hypothesized that the interaction between microglial autophagy and exosomes might be a new target for the treatment of cerebrovascular diseases.

### Autophagy and inflammation

Microglia, as the main source of inflammatory factors in CNS, are key mediators of neuroinflammation (Nayak et al., 2014; Colonna and Butovsky, 2017). The functional phenotype of microglia first followed the M1/M2 dichotomy of macrophages (Hu et al., 2015). Pro-inflammatory microglia are described as classically activated microglia (CAM), characterized by the secretion of destructive proinflammatory mediators such as ROS and proinflammatory cytokines, while anti-inflammatory microglia are described as alternative activated microglia (AAM), characterized by inflammatory resolution and tissue repair (Hu et al., 2015; Sikkema et al., 2018). However, this oversimplified classification that describes only two extreme states is outdated. In fact, microglia exhibit more dynamic and diverse functional phenotypes and there is no clear cut between the phenotypes (Ransohoff, 2016). Transcriptomic studies have shown that diverse functional phenotypes of microglia are determined by their transcriptional profiles (Song and Colonna, 2018), while microglia-mediated inflammatory responses are determined by their functional phenotypes. Numerous studies have found that microglial autophagy can regulate inflammatory responses by affecting their functional phenotypes in cerebrovascular diseases. Zhu et al. revealed that inhibition of the ER stress-autophagy axis could attenuate the deleterious activation of microglia in *in vivo* and *in vitro* studies, thereby inhibiting I/R-induced neuroinflammatory responses (Zhu et al., 2021). Wang et al. also demonstrated that inhibition of autophagy *via* Akt/mTOR pathway could attenuate OGD/R-induced inflammatory responses in BV2 microglia (Wang H. et al., 2020). However, He et al. (2020) found that restoring autophagic flux by suppressing mTOR could help shift the functional phenotypes of OGD/R-exposed BV2 microglia toward an anti-inflammatory phenotype. The different explanations may be related to multiple causes including the complex molecular networks between microglial autophagy and inflammatory responses. For instance, in addition to being a core molecule regulating autophagy, mTOR has been shown to play an important role in inflammatory responses of microglia in cerebrovascular diseases (Xie et al., 2014; Li D. et al., 2016).

Inflammasomes are intracellular protein complexes that serve as sensors for infective or traumatic stimuli and innate immune responses (Mangan et al., 2018). They are increasingly recognized as the core of the inflammatory responses. Numerous studies have shown that inflammasomes play an important role in various CNS diseases, particularly infectious diseases, cerebrovascular diseases, and neurodegenerative diseases (Walsh et al., 2014). They regulate the activity of inflammatory proteases in the caspase family by assembling cytoplasmic macromolecular complexes after infective or traumatic stimuli. The nod-like receptor protein 3 (NLRP3) inflammasome, as an important component of the inflammasome, has been demonstrated to have direct interaction with autophagy. Recent studies have shown that autophagy inhibits inflammasome activation by sequestering the NLRP3-binding protein ASC, which inhibits the activation of caspase-1 and the maturation of pro-IL-1β and pro-IL-18 (Shibutani et al., 2015). Subsequently, emerging data suggested that autophagy also negatively regulated NLRP3 inflammasome in microglia in pathological situations (Su et al., 2016), such as β-amyloid (Aβ)-mediated neuroinflammation *in vivo* (Cho et al., 2014) and ischemic-mediated neuroinflammation *in vitro* (Fu et al., 2020). It has been shown that activation of NLRP3 inflammasome is accompanied by impairment of autophagic function following cerebral ischemia *in vivo* studies. Besides, enhanced microglial autophagy has been demonstrated to suppress the activation of NLRP3 inflammasome in LPS-treated BV2 microglia (Espinosa-Garcia et al., 2020). Huang et al. also verified that enhanced autophagy *via* AMPK/mTOR/ULK1 pathway could suppress NLRP3 inflammasome-mediated neuroinflammation in LPS-treated BV2 microglia (Huang Z. et al., 2021). The therapeutic promise of inhibiting NLRP3 inflammasome activation by regulating microglial autophagy in cerebrovascular diseases has attracted increasing attention.

### Autophagy and phagocytosis

Microglial phagocytosis is a process of removing debris from healthy bodies, which can engulf apoptotic cells, myelin sheath, Aβ, and other substances under pathological conditions (Sierra et al., 2013). Microglial phagocytosis is considered an important part of its immune function and is critical for maintaining CNS homeostasis. On the one hand, microglial autophagy can affect the phagocytic function by modulating the activation of microglia; on the other hand, there are striking similarities in morphology and function between autophagy and phagocytosis. Both autophagy and phagocytosis are lysosomal degradation pathways that engulf and transport cargo *via* transient vesicular structures (autophagosomes and phagocytes, respectively) (Zang et al., 2019). In addition, autophagy and phagocytosis are the original forms of nutrient acquisition, and two processes play an important role in maintaining cell and tissue homeostasis through the degradation of intracellular and extracellular harmful substances, respectively. There is a complex network of interactions between the molecular mechanisms of autophagy and phagocytosis (Plaza-Zabala et al., 2017). LC3-associated phagocytosis (LAP), as a specialized kind of autophagy, demonstrates the cross-talk between autophagy and phagocytosis. Many autophagy-related proteins participate in LAP such as Beclin-1, Atg3, Atg5, and so on (Martinez, 2020 #363). LC3 is recruited to phagocytes with a monolayer membrane during LAP, which accelerates the maturation of phagosomes and promotes the fusion of phagosomes with lysosomes (Sanjuan et al., 2007). LAP is considered a non-canonical form of autophagy in microglia and plays an important role in the degradation of Aβ (Lee et al., 2019) and myelin (Berglund et al., 2020). Notably, it is necessary to distinguish autophagy or LAP when utilizing LC3 to refer to autophagosomes (Julg et al., 2020).

Inhibition of autophagy impairs the phagocytic capacity of microglia. The aforementioned study found that lipopolysaccharide (LPS) treatment significantly suppressed autophagic flux and expression of autophagy-related genes, which contributed to the impaired phagocytic capacity of microglia including LAP (Lee et al., 2019). Moreover, It has been demonstrated that Beclin-1 is required for efficient phagocytosis. Beclin-1-mediated phagocytic dysfunction was found to be associated with impaired recruitment of reverse transcriptase to the phagocytic membrane, decreased levels of reverse transcriptase, and impaired circulation of phagocytic receptors CD36 and Trem2 (Lucin et al., 2013). Impaired phagocytic function and detrimental effects on Aβ clearance of microglia in Alzheimer’s disease (AD) may due to this molecule mechanism (Lucin et al., 2013; O’Brien and Wyss-Coray, 2014). Nash et al. found that inhibition of autophagy reduced alpha-synuclein (α-Syn) phagocytosis by microglia (Nash et al., 2017). Induction of autophagy improves microglial phagocytosis, which may be the mechanism of the anti-neuroinflammatory effects of fluoxetine in stroke (Park et al., 2021). In conclusion, inhibition of autophagy leads to impaired phagocytic function of microglia, but the specific mechanism by which microglial autophagy regulates phagocytosis remains to be elucidated.

### Autophagy and metabolism

**FIGURE 2:**
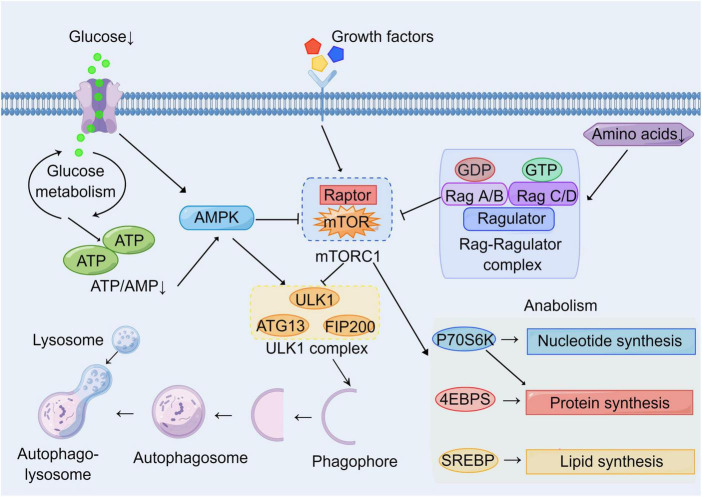
Molecular network of mTOR between autophagy and metabolism. Growth factors and energy levels are major upstream regulators of mTOR. Multiple growth factors can act on cell surface receptors to affect mTOR activation through various signaling pathways. Hypoglycemia and the reduction of ATP/AMP inhibit mTOR by activating AMPK. Low amino acid levels inhibit mTOR *via* the Rag-Ragulator complex pathway. Autophagy and metabolism are major downstream targets of mTOR. Activated mTOR inhibits nucleation and induction of phagophores by inhibiting the ULK1 complex. Meanwhile, activated mTOR promotes cell growth and proliferation by enhancing the synthesis of basal metabolites including proteins, nucleotides, and lipids through P70S6K, 4EBPs, and SREBP pathways.

Brain consumes large amounts of oxygen and has high energy requirements compared with other tissues or organs. It needs an adequate energy supply and stable metabolic pathways to maintain its normal physiological functions (Cunnane et al., 2020). Therefore, various acute and chronic CNS diseases such as stroke, TBI, and AD are generally accompanied by disturbances in metabolic pathways (Dusick et al., 2007; Ding et al., 2013; Robbins and Swanson, 2014; Gabbouj et al., 2019). For example, stroke leads to impaired cerebral blood circulation and hypoxia, resulting in impaired oxidative phosphorylation pathways and enhanced glycolysis pathways (Kim et al., 2006; Ham and Raju, 2017). AD is accompanied by metabolic disorders of Aβ and Tau, and is associated with insulin resistance (Gabbouj et al., 2019). Autophagy is a lysosomal degradation pathway to obtain energy and metabolic substrates. Therefore, autophagy is closely linked to metabolism, with mTOR being a key molecule linking cellular autophagy to metabolic networks (Perluigi et al., 2015). On the one hand, mTOR is regulated by glucose, amino acids, and cellular energy levels (AMP/ATP); on the other hand, the mTOR signal regulates downstream cellular metabolic pathways and autophagy induction (Laplante and Sabatini, 2012; Shimobayashi and Hall, 2014; Switon et al., 2017; Figure 2). In addition to mTOR, other autophagy-related proteins have also been found to be associated with cellular metabolic pathways. ULK1 can enhance glycolysis, reduce gluconeogenesis, and maintain intracellular ATP levels by directly acting on enzymes during glucose metabolism (Li T. Y. et al., 2016 #367). The above illustrates the complex network connection between autophagy and cellular metabolism.

Microglia have been found that the phenotypic transition of microglia may be mediated by cellular metabolic reprogramming, especially lipid and glucose metabolism (Lauro et al., 2019; Chausse et al., 2021). Single-cell RNA sequencing reveals a significant increase in the expression of some genes associated with lipid metabolism in the DAM phenotype, such as Trem2, Lpl, and ApoE (Keren-Shaul et al., 2017; Deczkowska et al., 2018; Benmamar-Badel et al., 2020). Li et al. found that enhanced glycolysis played an important role in the over-activation of hypoxia-exposed microglia in primary and BV2 microglia *in vitro*. And inhibition of enhanced glycolysis by knockdown of HK2 (a key rate-limiting enzyme of glycolysis) suppressed microglia-mediated neuroinflammatory responses, thereby reducing transient middle cerebral artery occlusion (MCAO) injury (Li et al., 2018). Song et al. found that microglia-specific knockout of NHE1 (a key PH regulator) shifted the microglial metabolic profile from glycolysis to oxidative phosphorylation, which enhanced microglial phagocytic activity and improved cognitive function following stroke (Song et al., 2022). It was also demonstrated that increasing oxidative phosphorylation-related gene expression and reducing glycolysis-related gene expression prevented the acquisition of a microglial pro-inflammatory phenotype in a permanent MCAO model (Lauro et al., 2019). However, little is known about the effect of microglial metabolism on autophagic function in cerebrovascular diseases.

Recent studies of Trem2-deficient mice in AD have revealed a link between microglial autophagy and cellular metabolism (Ulland et al., 2017; Zheng et al., 2018; Zhou et al., 2018). Trem2 is a surface receptor required by microglia in response to neurodegeneration, which correlates with microglial activation and metabolism. Trem2 can act directly on Aβ, thereby affecting the ability of microglia to respond to toxic amyloid plaques. Meanwhile, Trem2 can bind to apolipoproteins and lipoproteins, thereby regulating the uptake and metabolism of lipid in microglia. Subsequently, Trem2 can regulate microglial autophagic function as well as metabolic processes by activating the PI3K/Akt/mTOR pathway *via* DAP12 (Zheng et al., 2018). Studies have found that both mouse and human Trem2-deficient microglia exhibit impaired mTOR signaling, which leads to enhanced autophagy, decreased ATP levels, and impairment of biosynthetic metabolic pathways. Notably, incubation of Trem2-deficient microglia with cyclocreatine can produce a supply of ATP and rescue metabolic abnormalities, which ultimately reduces autophagy in microglia, suggesting that improving the metabolism of microglia can affect autophagic function (Ulland et al., 2017). Jia et al. found that the energy metabolism pathways of chronic Aβ-tolerant microglia were significantly impaired in the pathogenesis of AD, including oxidative phosphorylation and aerobic glycolysis. The defect in metabolism severely impairs the autophagic function of microglia and rescuing this metabolic defect can improve the autophagic function of microglia (Lu et al., 2021). Meanwhile, microglial autophagy can in turn affect their metabolic status. It has been found that cholesterol and neutral lipids accumulate in Atg7-deficient microglia, indicating that microglial autophagic function can affect their lipid metabolic homeostasis (Xu et al., 2021). Overall, the tight link between autophagic function and metabolic status of microglia, especially glucose and lipid metabolism, has been revealed in neurodegenerative diseases. The interaction between microglial metabolism and autophagy in cerebrovascular disease may become an expectant therapeutic target.

### Autophagy and neural circuit

It is well known that the functional remodeling of neural circuits is largely dependent on the autophagy and clearance mechanisms of cells. Microglia regulate synaptic remodeling, neurotransmission, and neuroplasticity by selectively degrading or adjusting pre- and post-synaptic components such as synaptic proteins, organelles, neurotransmitters, and their receptors (Paolicelli et al., 2011; Schafer et al., 2012). Cerebrovascular diseases are often thought to cause synapse dysfunction in formation or structure (Khatri and Man, 2013; Yan et al., 2021). Previously, an H_2_S donor, L-cysteine, has been shown to significantly alleviate brain damage after hypoxic-ischemic (HI) injury in neonatal mice. The protective effect was associated with enhanced autophagy flux of glia cells. L-cysteine attenuated HI induced synaptic damage and behavioral defects, and these effects were associated with up-regulation of synaptophysin and postsynaptic density protein-95 (PSD-95) in the damaged cortex (Xin et al., 2018). Similarly, studies have shown that minocycline reduced cognitive impairment by inhibiting mTOR signaling, enhancing autophagy process, and promoting the expression of presynaptic and PSD-95 in the brain of MCAO stroke rats (Wang S. et al., 2020). These above findings did not focus on some specific glial cells. However, Chen Man et al. first found that the autophagy process in microglia was involved in synaptic pruning. Microglia lacking ATG7 showed impaired synaptic degradation and increased number of immature synapses. Especially during early development, loss of autophagy in microglia impaired synaptic pruning and lead to increased dendritic spine density, which providing new insights into autism (Kim et al., 2017). More studies are still needed to further clarify the specific effects of microglial autophagy on neural circuits such as synaptic remodeling in cerebrovascular diseases.

### Effect of microglial autophagy in cerebrovascular diseases

**FIGURE 3:**
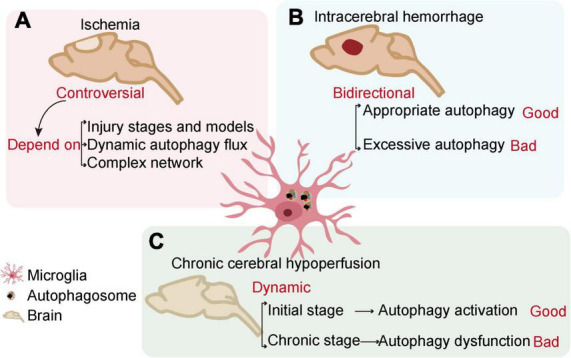
The relationship between microglial autophagy and cerebrovascular diseases. In ischemia **(A)**, intracerebral hemorrhage **(B)**, and chronic cerebral hypoperfusion **(C)**, microglial autophagy has different effects on the outcome of the diseases.

Cerebrovascular diseases, mainly caused by abnormal cerebral blood flow and secondary neurological injury, serve as major causes of death and disability worldwide. The common feature of cerebrovascular disease is to cause acute and chronic ischemia or hemorrhagic accidents in the brain (Moskowitz et al., 2010; Ferrer and Vidal, 2017). Microglia, as resident innate immune cells in the brain, are the main cells that respond to pathophysiological changes caused by cerebrovascular diseases. The interaction of microglial autophagy and other biological processes demonstrates the unique role of autophagy in microglial activation. Evidence has shown that the autophagic flux of microglia can play an important role in cerebrovascular diseases by regulating the phenotype and function of microglia (Su et al., 2016; Jiang et al., 2020). Next, we will discuss the role of microglial autophagy in ischemic stroke, intracerebral hemorrhage, and chronic cerebral hypoperfusion, respectively (Figure 3 and Table 1).

**TABLE 1:** List of outcomes after the interventions of autophagy in different cerebrovascular diseases.

Cerebrovascular diseases	Vivo/vitro	Patient/animal/cell	Model	Intervention	Target	Autophagy	Molecular mechanism	Outcome	References
Ischemia stroke	Vitro	Primary microglia	OGD	Hypoxia	Microglia	Induction	HIF-1α	Bad	Yang et al., 2014c
	Vitro	Brain microvascular endothelial cells (BMECs)	OGD/R	Hydroxysafflor yellow A	BMECs	Inhibition	PI3K/Akt/mTOR	Good	Yang et al., 2018
	Vivo/vitro	Rat/primary microglia	MCAO/LPS	SB216763	Microglia	Induction	GSK-3β	Good	Zhou et al., 2011
	Vivo/vitro	Mouse/primary microglia	PT/OGD	PARP14 overexpression	Microglia	Induction	LPAR5	Good	Tang et al., 2021
	Vitro	BV2	OGD/R	Hypoxia 24 h/72 h	Microglia	Induction/inhibition	NF-κB/CREB	Good/bad	Xia et al., 2016
	Vivo	Rat	MCAO	Excessive neuronal autophagy	Microglia	Induction	Cx3CL1-CX3CR1	Bad	He et al., 2019
	Vitro	BV2	OGD/R	Green Tea Catechin	Microglia	Inhibition	PI3K/Akt/mTOR	Good	Chen et al., 2016
	Vivo/vitro	Rat/primary astrocyte	MCAO/OGD	Autophagy inhibitor/siRNA	Astrocyte	Inhibition	tBID	Good	Zhou et al., 2017
Intracerebral hemorrhage	Vivo/vitro	Mouse/primary microglia	ICH/erythrocyte lysis	Autophagy inhibitor/siRNA	Microglia	Inhibition	/	Good	Yuan et al., 2017
	Vivo/vitro	Mouse/primary microglia	ICH/IL-17A	IL-17A	Microglia	Induction	/	Bad	Shi et al., 2018
	Vivo/vitro	Mouse/primary microglia	ICH/erythrocyte lysis	ICH/erythrocyte lysis	Microglia	Induction	TLR4	Bad	Yang et al., 2015a
	Vitro	Primary microglia	Hemoglobin	Hemoglobin	Microglia	Induction	miRNA-144/mTOR	Bad	Wang et al., 2017
	Vivo	Patient/mouse	ICH	PINK1	Microglia	Induction	PINK1	Good	Li G. et al., 2021
Chronic cerebral hypoperfusion	Vivo	Mouse	BCAS	Wortmannin	White matter	Inhibition	PI3K	Good	Zhang et al., 2019
	Vivo/vitro	Mouse/primary microglia	BCAS/LPS, IL-4	TLR4^–/–^	Microglia	Inhibition	STAT1/6	Good	Qin et al., 2018
	Vivo	Mouse	BCAS	3-MA	Microglia	Inhibition	PI3K	Good	Yang et al., 2014a
	Vivo	Rat	BCCAO	URB597	Microglia	Inhibition	ROS	Good	Su et al., 2019
	Vivo	Rat	BCCAO	Resveratrol	Neuron	Induction	Akt/mTOR	Good	Wang et al., 2019
	Vivo	Mouse	UCCAO	IMS-088	/	Induction	NF-κB	Good	Thammisetty et al., 2021
	Vivo/vitro	Rat/primary neuron	2VO	Antagomir-96	Hippocampus	Inhibition	mTOR	Good	Liu et al., 2018

OGD/R, Oxygen-Glucose Deprivation/Reoxygenation; MCAO, Middle Cerebral Artery Occlusion; LPS, Lipopolysaccharide; PT, Photothrombotic stroke; ICH, Intracerebral Hemorrhage; BCAS, Bilateral Carotid Artery Stenosis; BCCAO, Bilateral Common Carotid Artery Occlusion; UCCAO, Unilateral Common Carotid Artery Occlusion; 2VO, Two-Vessel Occlusions.

### Ischemia

Ischemic stroke is the most common and major cerebrovascular disease. As the most important pathophysiological feature of ischemic stroke, cerebral blood flow occlusion results in the deficiency of oxygen and glucose, ultimately leading to energy failure and metabolic disorders, as well as a series of secondary pathophysiological changes (Kunz et al., 2010 #368; Hossain et al., 2014; #369). Stimulators in ischemic areas such as ROS, extracellular ATP, and NO mediate the chemotaxis and activation of microglia (Tian et al., 2016 #370; Jia et al., 2022 #371; Li T. et al., 2022 #372). Reprogramming of the microglial metabolic profile mediates changes in the microglial phenotype, thereby affecting microglial immune function during cerebral ischemia. After cerebral ischemia, the mitochondrial oxidative phosphorylation pathway is impaired and the synthesis of ATP is reduced in microglia. The enhanced glycolytic pathway occupies a dominant position in the glucose metabolism of microglia, which activates the proinflammatory phenotype of microglia (Li et al., 2018; Lauro et al., 2019). At the same time, the lipid and lipoprotein metabolic profiles, especially the fatty acid oxidation pathways, play an important role in the phenotypic changes of microglia (Liao et al., 2020; Yang et al., 2021). Under stressful conditions of ischemia, a metabolic state with low energy levels enhances microglial autophagy to degrade excess proteins and organelles, which produces sufficient nutrients and energy. Microglia autophagy can modulate microglial activation and function, thereby affecting neuroinflammatory responses during cerebral ischemia (He et al., 2020; Zhu et al., 2021; Lin et al., 2022).

Traditionally, autophagy has a protective effect on ischemic brain tissues. Studies have shown that hyperbaric oxygen preconditioning or sublethal ischemic preconditioning enhanced autophagy activity, leading to the neuroprotective effect of cerebral ischemia injury (Yan et al., 2011; Mo et al., 2020). Several intracellular molecules such as PARP14 (a member in the Poly (ADP-ribose) polymerase superfamily) and PGC-1α (peroxisome proliferator-activated receptor-γ coactivator-1α) have been demonstrated to limit microglial activation and promote neurological recovery after stroke by inducing microglial autophagy (Han et al., 2021; Tang et al., 2021). Some drugs such as SB216763 (the serine/threonine kinase GSK-3β inhibitor) and Pien-Tze-Huang (a patent formula in the Chinese Pharmacopoeia for the treatment of inflammatory diseases) have also been found to suppress microglial over-activation and inflammatory response by inducing microglial autophagy in ischemic stroke (Zhou et al., 2011; Huang Z. et al., 2021; Tang et al., 2021). Xia et al. found that following OGD/R injury, blocking autophagic flux induced the transition of BV2 microglia toward a pro-inflammatory phenotype, which increases the release of proinflammatory cytokines and contributes to neuroinflammation (Xia et al., 2016). However, increasing evidence shows that microglial autophagy induced by ischemia aggravates neuroinflammation and injury and autophagy inhibition is beneficial for ischemia (Yang et al., 2015b; Zhu et al., 2021). The autophagy inhibitor (3-MA) supports this idea. 3-MA inhibits the induction of autophagy *via* PI3K and significantly alleviates the infarct area, edema formation and nerve functional defect in ischemic stroke (Yang et al., 2015b; He et al., 2019). Studies on microglial autophagy in ischemia got different conclusions. The explanation for these differences may be related to a number of reasons. First, the autophagic flux of BV2 microglia changes at different stages of OGD/R injury. In the early phase of reoxygenation, autophagic flux of microglia is induced while in the late phase of reoxygenation, the autophagic flux is inhibited, which leads to the alternation of microglial phenotypes during ischemia (Xia et al., 2016). Second, the level of autophagic flux in microglia is difficult to determine. And then, there are complex molecular networks between microglial autophagy and functional phenotypes (section “Autophagy and exosomes”). Finally, these studies involve different animal models, cell types, and treatments. In conclusion, microglia autophagy mainly plays an important role in ischemic stroke by regulating the microglial phenotypes and inflammation responses. And it is generally accepted that regulating microglial autophagy may become a new insight into the treatment strategies for ischemic stroke.

### Intracerebral hemorrhage

Intracerebral hemorrhage (ICH) has higher mortality compared with ischemia (Krishnamurthi et al., 2014 #373). Blood–brain barrier (BBB) disruption is the main pathophysiological change of ICH, leading to hematoma formation and cerebral edema, accompanied by peripheral blood circulation disorders, metabolic disorders, and secondary neuroinflammatory damage (Wang, 2010; Yang et al., 2014b). Besides, leaky blood components such as thrombin, heme, hemoglobin, and cell debris can also cause a secondary inflammatory response, which is characterized by the accumulation and activation of inflammatory cells including microglia and the release of proinflammatory mediators (Shi et al., 2018). The secondary inflammatory response is critical for the prognosis of ICH. Reduction of pro-inflammatory mediators provides a potentially effective approach to ICH therapy. Therefore, we focus on the role of microglial autophagy on the inflammatory response in ICH.

Previous studies have shown that ICH motivated microglial autophagy. Autophagy inhibition by drug inhibitors or RNA interference with the necessary autophagy genes (BECN1 and ATG5) decreased activation and inflammatory response of microglia (Yuan et al., 2017). Shi et al. (2018) also found that IL-17A, an inflammatory mediator in ICH, induced microglial autophagy through ATG5 and ATG7, which promoted the inflammatory response of microglia and inflammatory damage to neurons. It has been reported that the erythrocyte lysates after ICH promotes microglial autophagy *via* TLR4, which results in microglial activation and promotes inflammatory response. Both autophagy inhibitor (3-MA) and TLR4^–/–^ mice significantly reduced cerebral edema and neurological injury in ICH compared with WT mice (Yang et al., 2015a). In addition, Wang et al. found that miRNAs were key factors in autophagy, which negatively and post-transcriptionally regulated gene expression and function. After ICH, leaky hemoglobin promoted the expression of miRNA-144. MiRNA-144 has been involved in hemoglobin-mediated microglial autophagic induction and inflammatory response through the mTOR pathway, which exacerbated neurological symptoms and led to brain function impairment after ICH (Wang et al., 2017). Recent studies have found that PINK1, a key enzyme in mitochondria, can promote mitophagy in microglia to protect against ICH-induced brain injury (Li J. et al., 2021). In summary, Microglial autophagy presents a bidirectional role in ICH. Appropriate activation of mitophagy facilitates maintenance of energy homeostasis in microglia, while excessive autophagy leads to over-activation of microglia, exacerbating the neuroinflammatory response.

### Chronic cerebral hypoperfusion

Chronic cerebral hypoperfusion is a process of long-term cerebral ischemia and hypoxia caused by a variety of etiologies, and its typical pathological features are demyelination, axonal injury, and white matter lesions (Shang et al., 2019). These pathological features lead to cognitive dysfunction and even vascular dementia, which has become the second leading cause of dementia after AD (Rizzi et al., 2014). The specific mechanism of chronic cerebral hypoperfusion remains unclear. In the past, neuroinflammatory response was considered to be a critical pathogenetic factor. Ischemia and hypoxia during chronic cerebral hypoperfusion lead to mitochondrial dysfunction, cellular metabolic energy failure and increased levels of oxidative stress, thereby exacerbating the neuroinflammatory response in the brain (Zhao et al., 2021 #375). Meanwhile, microglia significantly transform into an proinflammatory phenotype and release proinflammatory cytokines, thereby causing white matter injury in the brain (Urabe, 2012). Recent studies suggest that autophagy also plays an important role in the vascular pathology of chronic cerebral hypoperfusion (Wang X. X. et al., 2020). Cerebral hypoperfusion and impaired blood-brain barrier clearance result in the accumulation of a series of neurotoxic metabolites in the hypoperfusion area, especially Aβ (Zlokovic, 2008) and myelin debris (Zheng et al., 2022). Therefore, based on the important role of microglial LAP in clearance and degradation of Aβ and myelin debris (section “Autophagy and inflammation”), we hypothesize that in addition to affecting functional phenotypes of microglia (Yang et al., 2014a), microglial autophagy acts in the form of LAP in chronic cerebral hypoperfusion.

Our previous studies have shown that autophagic activity in white matter is activated early after chronic hypoperfusion in a mouse model of bilateral common carotid artery stenosis (BCAS), and it is suppressed slightly in the mid-ischemic phase and reactivated late after ischemia (Zhang et al., 2019). Besides, multiple studies have also demonstrated that the autophagic activity of neurons or microglia is activated following chronic cerebral hypoperfusion (Qin et al., 2018; Liu et al., 2019). The autophagy inhibitor (3-MA) can reduce white matter injury caused by chronic cerebral hypoperfusion by suppressing microglial autophagy (Yang et al., 2014a). We have previously shown that TLR4-dependent autophagy induces ischemic white matter damage by promoting the over-activation of microglia. Autophagy inhibitors wortmannin and bafilomycin A1 inhibit autophagy by reducing the induction of autophagy and blocking autophagic flux, respectively. They decrease the expression of microglial pro-inflammatory transcripts such as CD86, INOS, TNF-α, and IL-β, switching microglia to anti-inflammatory phenotype (Qin et al., 2018). Su et al. found that over-activation and pro-inflammatory phenotype of microglia may be upstream regulators of chronic cerebral hypoperfusion induced autophagy *in vivo* studies (Su et al., 2019). Therefore, the causal relationship between microglial autophagy and functional phenotypes is in dynamic changes during chronic cerebral hypoperfusion. At the initial stage of cerebral hypoperfusion, enhanced autophagy can exert neuroprotective effects. With prolonged ischemia and hypoxia, the exacerbated neuroinflammatory response leads to autophagy dysfunction, which in turn acts on microglia to further aggravate the neuroinflammatory response.

## Conclusion and future perspectives

This review presents a comprehensive introduction to the interaction between microglial autophagy and other biological processes, especially metabolism, in physiological and pathological situations, and focuses on microglial autophagy in cerebrovascular diseases. Different studies have provided different evidence for the effects of autophagy on various biological processes of microglia such as apoptosis, phagocytosis, and inflammation. Microglial autophagy promotes the phagocytosis and degradation of pathogenic substances such as Aβ and α-Syn, which delays the progression of primary neurodegenerative diseases such as AD and PD (Nash et al., 2017 #182, Lee et al., 2019 #168). And microglial autophagy promotes the clearance of myelin debris, which contributes to remyelination and nerve repair in demyelinating diseases (Berglund et al., 2020 #184). In cerebrovascular diseases, ischemic stroke leads to metabolic changes and structural damage in cells, resulting in cellular acidosis, mitochondrial ROS generation, and intracellular calcium overload (Mo et al., 2020); disruption of the BBB in ICH triggers a series of pathological reactions, such as the formation of cerebral hematoma, the cascade inflammatory response of erythrocyte lysates and so on (Yang et al., 2015a). The current studies show that microglial autophagy presents a complex and bidirectional role in various cerebrovascular diseases, which may be related to the type of cerebrovascular diseases, the progression of the diseases, and the choices of experimental models. Besides, it is necessary to accurately judge the changes of autophagy in experiments since there is no universal gold standard. Overall, microglial autophagy occupies an important position in the pathogenesis of cerebrovascular disease and is closely associated with many biological processes, particularly the inflammatory response and metabolic state of microglia.

Inspired by studies of the role of microglial autophagy in cerebrovascular diseases, research on the treatment of cerebrovascular disease through this pathway has recently begun to emerge. Resveratrol inhibits NLRP3 inflammasome activation by enhancing autophagy, thereby exerting neuroprotective effects in I/R injury (He et al., 2017). Progesterone has also been found to attenuate neuroinflammatory responses and improve prognosis after cerebral ischemia by inhibiting stress-induced activation of NLRP3 inflammasome, which is mediated by enhancing autophagy (Espinosa-Garcia et al., 2020). Recent studies have found that microglial ER stress-autophagy axis can also become the target of treatment in ischemic stroke (Zhu et al., 2021). Microglial autophagy has a positive research prospect as a new target for the treatment of cerebrovascular diseases. However, it must be emphasized that the role of microglial autophagy in the pathogenetic mechanism and post-injury repair of cerebrovascular diseases is not clear and requires more detailed studies. A comprehensive understanding of the key role of microglial autophagy in cerebrovascular diseases will provide a novel direction for finding new therapeutic agents for cerebrovascular diseases.

## Author contributions

MC conceived the idea of this review. MC and HZ drafted the manuscript and created the figures. Y-HC, YT, and X-WP performed the literature search and reviewed the content of this manuscript. All authors read and approved the final manuscript.

## References

[B1] Anderson S. R. Roberts J. M. Zhang J. Steele M. R. Romero C. O. Bosco A. (2019). Developmental apoptosis promotes a disease-related gene signature and independence from CSF1R signaling in retinal microglia. *Cell Rep.* 27 2002.e5–2013.e5. 10.1016/j.celrep.2019.04.062 31091440 PMC6544177

[B2] Andoh M. Koyama R. (2021). Comparative review of microglia and monocytes in CNS phagocytosis. *Cells* 10:2555. 10.3390/cells10102555 34685535 PMC8534258

[B3] Benmamar-Badel A. Owens T. Wlodarczyk A. (2020). Protective microglial subset in development, aging, and disease: lessons from transcriptomic studies. *Front. Immunol.* 11:430. 10.3389/fimmu.2020.00430 32318054 PMC7147523

[B4] Berglund R. Guerreiro-Cacais A. O. Adzemovic M. Z. Zeitelhofer M. Lund H. Ewing E. (2020). Microglial autophagy-associated phagocytosis is essential for recovery from neuroinflammation. *Sci. Immunol.* 5:eabb5077. 10.1126/sciimmunol.abb5077 33067381

[B5] Chausse B. Kakimoto P. A. Kann O. (2021). Microglia and lipids: how metabolism controls brain innate immunity. *Semin. Cell Dev. Biol.* 112 137–144. 10.1016/j.semcdb.2020.08.001 32807643

[B6] Chen C.-M. Wu C.-T. Yang T.-H. Chang Y.-A. Sheu M.-L. Liu S. H. (2016). Green tea catechin prevents hypoxia/reperfusion-evoked oxidative stress-regulated autophagy-activated apoptosis and cell death in microglial cells. *J. Agric. Food Chem.* 64 4078–4085. 10.1021/acs.jafc.6b01513 27144449

[B7] Cho M.-H. Cho K. Kang H.-J. Jeon E.-Y. Kim H.-S. Kwon H.-J. (2014). Autophagy in microglia degrades extracellular beta-amyloid fibrils and regulates the NLRP3 inflammasome. *Autophagy* 10 1761–1775. 10.4161/auto.29647 25126727 PMC4198361

[B8] Colonna M. Butovsky O. (2017). Microglia function in the central nervous system during health and neurodegeneration. *Annu. Rev. Immunol.* 35 441–468. 28226226 10.1146/annurev-immunol-051116-052358 PMC8167938

[B9] Cunnane S. C. Trushina E. Morland C. Prigione A. Casadesus G. Andrews Z. B. (2020). Brain energy rescue: an emerging therapeutic concept for neurodegenerative disorders of ageing. *Nat. Rev. Drug Discov.* 19 609–633. 10.1038/s41573-020-0072-x 32709961 PMC7948516

[B10] Deczkowska A. Keren-Shaul H. Weiner A. Colonna M. Schwartz M. Amit I. (2018). Disease-associated microglia: a universal immune sensor of neurodegeneration. *Cell* 173 1073–1081. 10.1016/j.cell.2018.05.003 29775591

[B11] Deng W. Mandeville E. Terasaki Y. Li W. Holder J. Chuang A. T. (2020). Transcriptomic characterization of microglia activation in a rat model of ischemic stroke. *J. Cereb. Blood Flow Metab.* 40 S34–S48. 10.1177/0271678X20932870 33208001 PMC7687036

[B12] Diaz-Aparicio I. Paris I. Sierra-Torre V. Plaza-Zabala A. Rodriguez-Iglesias N. Marquez-Ropero M. (2020). Microglia actively remodel adult hippocampal neurogenesis through the phagocytosis secretome. *J. Neurosci.* 40 1453–1482. 10.1523/JNEUROSCI.0993-19.2019 31896673 PMC7044727

[B13] Ding F. Yao J. Rettberg J. R. Chen S. Brinton R. D. (2013). Early decline in glucose transport and metabolism precedes shift to ketogenic system in female aging and Alzheimer’s mouse brain: implication for bioenergetic intervention. *PLoS One* 8:e79977. 10.1371/journal.pone.0079977 24244584 PMC3823655

[B14] Ding Z. B. Han Q. X. Wang Q. Song L. J. Chu G. G. Guo M. F. (2021). Fasudil enhances the phagocytosis of myelin debris and the expression of neurotrophic factors in cuprizone-induced demyelinating mice. *Neurosci. Lett.* 753:135880. 10.1016/j.neulet.2021.135880 33838256

[B15] Dusick J. R. Glenn T. C. Lee W. N. Vespa P. M. Kelly D. F. Lee S. M. (2007). Increased pentose phosphate pathway flux after clinical traumatic brain injury: a [1,2-13C2]glucose labeling study in humans. *J. Cereb. Blood Flow Metab.* 27 1593–1602. 10.1038/sj.jcbfm.9600458 17293841

[B16] Espinosa-Garcia C. Atif F. Yousuf S. Sayeed I. Neigh G. N. Stein D. G. (2020). Progesterone attenuates stress-induced NLRP3 inflammasome activation and enhances autophagy following ischemic brain injury. *Int. J. Mol. Sci.* 21:3740. 10.3390/ijms21113740 32466385 PMC7312827

[B17] Estfanous S. Daily K. P. Eltobgy M. Deems N. P. Anne M. N. K. Krause K. (2021). Elevated expression of MiR-17 in microglia of Alzheimer’s disease patients abrogates autophagy-mediated amyloid-beta degradation. *Front. Immunol.* 12:705581. 10.3389/fimmu.2021.705581 34426734 PMC8379081

[B18] Fader C. M. Colombo M. I. (2006). Multivesicular bodies and autophagy in erythrocyte maturation. *Autophagy* 2 122–125. 10.4161/auto.2.2.2350 16874060

[B19] Fader C. M. Sanchez D. Furlan M. Colombo M. I. (2008). Induction of autophagy promotes fusion of multivesicular bodies with autophagic vacuoles in K562 cells. *Traffic* 9 230–250. 10.1111/j.1600-0854.2007.00677.x 17999726

[B20] Feng D. Wang B. Wang L. Abraham N. Tao K. Huang L. (2017). Pre-ischemia melatonin treatment alleviated acute neuronal injury after ischemic stroke by inhibiting endoplasmic reticulum stress-dependent autophagy via PERK and IRE1 signalings. *J. Pineal Res.* 62:e12395. 10.1111/jpi.12395 28178380

[B21] Ferrer I. Vidal N. (2017). Neuropathology of cerebrovascular diseases. *Handb. Clin. Neurol.* 145 79–114. 10.1016/B978-0-12-802395-2.00007-9 28987197

[B22] Fu C. Zhang X. Lu Y. Wang F. Xu Z. Liu S. (2020). Geniposide inhibits NLRP3 inflammasome activation via autophagy in BV-2 microglial cells exposed to oxygen-glucose deprivation/reoxygenation. *Int. Immunopharmacol.* 84:106547. 10.1016/j.intimp.2020.106547 32361652

[B23] Gabbouj S. Ryhanen S. Marttinen M. Wittrahm R. Takalo M. Kemppainen S. (2019). Altered insulin signaling in Alzheimer’s disease brain - special emphasis on PI3K-Akt pathway. *Front. Neurosci.* 13:629. 10.3389/fnins.2019.00629 31275108 PMC6591470

[B24] Ghavami S. Shojaeid S. Yeganeh B. Ande S. R. Jangamreddy J. R. Mehrpour M. (2014). Autophagy and apoptosis dysfunction in neurodegenerative disorders. *Prog. Neurobiol.* 112 24–49. 10.1016/j.pneurobio.2013.10.004 24211851

[B25] Ginhoux F. Greter M. Leboeuf M. Nandi S. See P. Gokhan S. (2010). Fate mapping analysis reveals that adult microglia derive from primitive macrophages. *Science* 330 841–845. 10.1126/science.1194637 20966214 PMC3719181

[B26] Guo M. L. Buch S. (2019). Neuroinflammation & pre-mature aging in the context of chronic HIV infection and drug abuse: role of dysregulated autophagy. *Brain Res.* 1724:146446. 10.1016/j.brainres.2019.146446 31521638 PMC6933726

[B27] Ham P. B. III Raju R. (2017). Mitochondrial function in hypoxic ischemic injury and influence of aging. *Prog. Neurobiol.* 157 92–116. 10.1016/j.pneurobio.2016.06.006 27321753 PMC5161736

[B28] Han B. Jiang W. Cui P. Zheng K. Dang C. Wang J. (2021). Microglial PGC-1alpha protects against ischemic brain injury by suppressing neuroinflammation. *Genome Med.* 13:47. 10.1186/s13073-021-00863-5 33771213 PMC8004413

[B29] He C. Klionsky D. J. (2009). Regulation mechanisms and signaling pathways of autophagy. *Annu. Rev. Genet.* 43 67–93. 10.1146/annurev-genet-102808-114910 19653858 PMC2831538

[B30] He H.-Y. Ren L. Guo T. Deng Y.-H. (2019). Neuronal autophagy aggravates microglial inflammatory injury by downregulating CX3CL1/fractalkine after ischemic stroke. *Neural Regener. Res.* 14 280–288. 10.4103/1673-5374.244793 30531011 PMC6301168

[B31] He Q. Li Z. Wang Y. Hou Y. Li L. Zhao J. (2017). Resveratrol alleviates cerebral ischemia/reperfusion injury in rats by inhibiting NLRP3 inflammasome activation through Sirt1-dependent autophagy induction. *Int. Immunopharmacol.* 50 208–215. 10.1016/j.intimp.2017.06.029 28683365

[B32] He T. Li W. Song Y. Li Z. Tang Y. Zhang Z. (2020). Sestrin2 regulates microglia polarization through mTOR-mediated autophagic flux to attenuate inflammation during experimental brain ischemia. *J. Neuroinflamm.* 17:329. 10.1186/s12974-020-01987-y 33153476 PMC7643276

[B33] Hossain M. I. Roulston C. L. Stapleton D. I. (2014). Molecular basis of impaired glycogen metabolism during ischemic stroke and hypoxia. *PLoS One* 9:e97570. 10.1371/journal.pone.0097570 24858129 PMC4032261

[B34] Hu X. Leak R. K. Shi Y. Suenaga J. Gao Y. Zheng P. (2015). Microglial and macrophage polarization-new prospects for brain repair. *Nat. Rev. Neurol.* 11 56–64. 10.1038/nrneurol.2014.207 25385337 PMC4395497

[B35] Hu Z. Yuan Y. Zhang X. Lu Y. Dong N. Jiang X. (2021). Human umbilical cord mesenchymal stem cell-derived exosomes attenuate oxygen-glucose deprivation/reperfusion-induced microglial pyroptosis by promoting FOXO3a-dependent mitophagy. *Oxid. Med. Cell Longev.* 2021:6219715. 10.1155/2021/6219715 34765084 PMC8577931

[B36] Huang T. Lin Y. Pang Q. Shen W. Chen X. Tu F. (2021). The synergistic effect of TRPV1 on oxidative stress-induced autophagy and apoptosis in microglia. *Anal. Cell Pathol.* 2021:7955791. 10.1155/2021/7955791 34336554 PMC8298174

[B37] Huang Z. Zhou X. Zhang X. Huang L. Sun Y. Cheng Z. (2021). Pien-Tze-Huang, a Chinese patent formula, attenuates NLRP3 inflammasome-related neuroinflammation by enhancing autophagy via the AMPK/mTOR/ULK1 signaling pathway. *Biomed. Pharmacother.* 141:111814. 10.1016/j.biopha.2021.111814 34146853

[B38] Jia X. Xie L. Liu Y. Liu T. Yang P. Hu J. (2022). Astragalus polysaccharide (APS) exerts protective effect against acute ischemic stroke (AIS) through enhancing M2 micoglia polarization by regulating adenosine triphosphate (ATP)/purinergic receptor (P2X7R) axis. *Bioengineered*. 13, 4468–4480. 10.1080/21655979.2021.1980176 35166175 PMC8973874

[B39] Jiang C. T. Wu W. F. Deng Y. H. Ge J. W. (2020). Modulators of microglia activation and polarization in ischemic stroke (Review). *Mol. Med. Rep.* 21 2006–2018. 10.3892/mmr.2020.11003 32323760 PMC7115206

[B40] Jiang M. Wang H. Zin M. Yang X. Ji H. Jiang Y. (2018). Exosomes from MiR-30d-5p-ADSCs reverse acute ischemic stroke-induced, autophagy-mediated brain injury by promoting M2 microglial/macrophage polarization. *Cell. Physiol. Biochem.* 47 864–878. 10.1159/000490078and 29807362

[B41] Jin Y. Wang R. Yang S. Zhang X. Dai J. (2017). Role of microglia autophagy in microglia activation after traumatic brain injury. *World Neurosurg.* 100 351–360. 10.1016/j.wneu.2017.01.033 28108422

[B42] Julg J. Strohm L. Behrends C. (2020). Canonical and non-canonical autophagy pathways in microglia. *Mol. Cell Biol.* 41:e00389-20. 10.1128/MCB.00389-20 33139495 PMC8088277

[B43] Keren-Shaul H. Spinrad A. Weiner A. Matcovitch-Natan O. Dvir-Szternfeld R. Ulland T. K. (2017). A unique microglia type associated with restricting development of Alzheimer’s disease. *Cell* 169 1276.e17–1290.e17. 10.1016/j.cell.2017.05.018 28602351

[B44] Khatri N. Man H.-Y. (2013). Synaptic activity and bioenergy homeostasis: implications in brain trauma and neurodegenerative diseases. *Front. Neurol.* 4:199. 10.3389/fneur.2013.00199 24376435 PMC3858785

[B45] Kim H. J. Cho M. H. Shim W. H. Kim J. K. Jeon E. Y. Kim D. H. (2017). Deficient autophagy in microglia impairs synaptic pruning and causes social behavioral defects. *Mol. Psychiatry* 22 1576–1584. 10.1038/mp.2016.103 27400854 PMC5658669

[B46] Kim J. W. Tchernyshyov I. Semenza G. L. Dang C. V. (2006). HIF-1-mediated expression of pyruvate dehydrogenase kinase: a metabolic switch required for cellular adaptation to hypoxia. *Cell Metab.* 3 177–185. 10.1016/j.cmet.2006.02.002 16517405

[B47] Klionsky D. J. (2005). The molecular machinery of autophagy: unanswered questions. *J. Cell Sci.* 118(Pt 1), 7–18. 10.1242/jcs.01620 15615779 PMC1828869

[B48] Krasemann S. Madore C. Cialic R. Baufeld C. Calcagno N. El Fatimy R. (2017). The TREM2-APOE pathway drives the transcriptional phenotype of dysfunctional microglia in neurodegenerative diseases. *Immunity* 47 566.e9–581.e9. 10.1016/j.immuni.2017.08.008 28930663 PMC5719893

[B49] Krishnamurthi R. V. Moran A. E. Forouzanfar M. H. Bennett D. A. Mensah G. A. Lawes C. M. (2014). The global burden of hemorrhagic stroke: a summary of findings from the GBD 2010 study. *Glob. Heart.* 9, 101–106. 10.1016/j.gheart.2014.01.003 25432119

[B50] Kunz A. Dirnagl U. Mergenthaler P. (2010). Acute pathophysiological processes after ischaemic and traumatic brain injury. *Best. Pract. Res. Clin. Anaesthesiol.* 24, 495–509. 10.1016/j.bpa.2010.10.001 21619862

[B51] Ladeby R. Wirenfeldt M. Garcia-Ovejero D. Fenger C. Dissing-Olesen L. Dahnau I. (2005). Microglial cell population dynamics in the injured adult central nervous system. *Brain Res. Rev.* 48 196–206. 10.1016/j.brainresrev.2004.12.009 15850658

[B52] Laplante M. Sabatini D. M. (2012). mTOR signaling in growth control and disease. *Cell* 149 274–293. 10.1016/j.cell.2012.03.017 22500797 PMC3331679

[B53] Lauro C. Chece G. Monaco L. Antonangeli F. Peruzzi G. Rinaldo S. (2019). Fractalkine modulates microglia metabolism in brain ischemia. *Front. Cell Neurosci.* 13:414. 10.3389/fncel.2019.00414 31607865 PMC6755341

[B54] Lee J.-W. Nam H. Kim L. E. Jeon Y. Min H. Ha S. (2019). TLR4 (toll-like receptor 4) activation suppresses autophagy through inhibition of FOXO3 and impairs phagocytic capacity of microglia. *Autophagy* 15 753–770. 10.1080/15548627.2018.1556946 30523761 PMC6526818

[B55] Levine B. Kroemer G. (2019). Biological functions of autophagy genes: a disease perspective. *Cell* 176 11–42. 10.1016/j.cell.2018.09.048 30633901 PMC6347410

[B56] Levine B. Mizushima N. Virgin H. W. (2011). Autophagy in immunity and inflammation. *Nature* 469 323–335. 10.1038/nature09782 21248839 PMC3131688

[B57] Li D. Wang C. Yao Y. Chen L. Liu G. Zhang R. (2016). mTORC1 pathway disruption ameliorates brain inflammation following stroke via a shift in microglia phenotype from M1 type to M2 type. *FASEB J.* 30 3388–3399. 10.1096/fj.201600495R 27342766

[B58] Li G. Sherchan P. Tang Z. Tang J. (2021). Autophagy & phagocytosis in neurological disorders and their possible cross-talk. *Curr. Neuropharmacol.* 19 1912–1924. 10.2174/1570159X19666210407150632 33827410 PMC9185789

[B59] Li J. Wu X. He Y. Wu S. Guo E. Feng Y. (2021). PINK1 antagonize intracerebral hemorrhage by promoting mitochondrial autophagy. *Ann. Clin. Transl. Neurol.* 8 1951–1960. 10.1002/acn3.51425 34453779 PMC8528457

[B60] Li P. Luo X. Luo Z. He G. L. Shen T. T. Yu X. T. (2022). Increased miR-155 in microglial exosomes following heat stress accelerates neuronal autophagy via their transfer into neurons. *Front. Cell Neurosci.* 16:865568. 10.3389/fncel.2022.865568 35634460 PMC9132214

[B61] Li Q. Barres B. A. (2018). Microglia and macrophages in brain homeostasis and disease. *Nat. Rev. Immunol.* 18 225–242. 10.1038/nri.2017.125 29151590

[B62] Li T. Xu T. Zhao J. Gao H. Xie W. (2022). Depletion of iNOS-positive inflammatory cells decelerates neuronal degeneration and alleviates cerebral ischemic damage by suppressing the inflammatory response. *Free Radic. Biol. Med.* 181, 209–220. 10.1016/j.freeradbiomed.2022.02.008 35150825

[B63] Li T. Y. Sun Y. Liang Y. Liu Q. Shi Y. Zhang C. S. (2016). ULK1/2 constitute a bifurcate node controlling glucose metabolic fluxes in addition to autophagy. *Mol. Cell*. 62, 359–370. 10.1016/j.molcel.2016.04.009 27153534

[B64] Li X. Li K. Chu F. Huang J. Yang Z. (2020). Graphene oxide enhances beta-amyloid clearance by inducing autophagy of microglia and neurons. *Chem. Biol. Interact.* 325:109126. 10.1016/j.cbi.2020.109126 32430275

[B65] Li Y. Lu B. Sheng L. Zhu Z. Sun H. Zhou Y. (2018). Hexokinase 2-dependent hyperglycolysis driving microglial activation contributes to ischemic brain injury. *J. Neurochem.* 144 186–200. 10.1111/jnc.14267 29205357

[B66] Liao B. Geng L. Zhang F. Shu L. Wei L. Yeung P. K. K. (2020). Adipocyte fatty acid-binding protein exacerbates cerebral ischaemia injury by disrupting the blood-brain barrier. *Eur. Heart J.* 41 3169–3180. 10.1093/eurheartj/ehaa207 32350521 PMC7556749

[B67] Lin Y. Huang T. Shen W. Pang Q. Xie Q. Chen X. (2022). TRPV1 suppressed NLRP3 through regulating autophagy in microglia after ischemia-reperfusion injury. *J. Mol. Neurosci.* 72 792–801. 10.1007/s12031-021-01935-2 35041191

[B68] Liu B. Liu J. Zhang J. Mao W. Li S. (2019). Effects of autophagy on synaptic-plasticity-related protein expression in the hippocampus CA1 of a rat model of vascular dementia. *Neurosci. Lett.* 707:134312. 10.1016/j.neulet.2019.134312 31163225

[B69] Liu P. Liu P. Wang Z. Fang S. Liu Y. Wang J. (2018). Inhibition of MicroRNA-96 ameliorates cognitive impairment and inactivation autophagy following chronic cerebral hypoperfusion in the rat. *Cell Physiol. Biochem.* 49 78–86. 10.1159/000492844 30134226

[B70] Lu J. Zhou W. Dou F. Wang C. Yu Z. (2021). TRPV1 sustains microglial metabolic reprogramming in Alzheimer’s disease. *EMBO Rep.* 22:e52013. 10.15252/embr.202052013 33998138 PMC8183394

[B71] Lucin K. M. O’Brien C. E. Bieri G. Czirr E. Mosher K. I. Abbey R. J. (2013). Microglial beclin 1 regulates retromer trafficking and phagocytosis and is impaired in Alzheimer’s disease. *Neuron* 79 873–886. 10.1016/j.neuron.2013.06.046 24012002 PMC3779465

[B72] Lum J. J. DeBerardinis R. J. Thompson C. B. (2005). Autophagy in metazoans: cell survival in the land of plenty. *Nat. Rev. Mol. Cell Biol.* 6 439–448. 10.1038/nrm1660 15928708

[B73] Maiuri M. C. Zalckvar E. Kimchi A. Kroemer G. (2007). Self-eating and self-killing: crosstalk between autophagy and apoptosis. *Nat. Rev. Mol. Cell Biol.* 8 741–752. 10.1038/nrm2239 17717517

[B74] Mangan M. S. J. Olhava E. J. Roush W. R. Seidel H. M. Glick G. D. Latz E. (2018). Targeting the NLRP3 inflammasome in inflammatory diseases. *Nat. Rev. Drug Discov.* 17 588–606. 10.1038/nrd.2018.97 30026524

[B75] Martinez J. (2020). Detection of LC3-associated phagocytosis (LAP). *Curr. Protoc. Cell Biol.* 87. 10.1002/cpcb.104 32436654 PMC9285819

[B76] Mo Y. Sun Y.-Y. Liu K.-Y. (2020). Autophagy and inflammation in ischemic stroke. *Neural Regener. Res.* 15 1388–1396. 10.4103/1673-5374.274331 31997797 PMC7059569

[B77] Moskowitz M. A. Lo E. H. Iadecola C. (2010). The science of stroke: mechanisms in search of treatments. *Neuron* 67 181–198. 10.1016/j.neuron.2010.07.002 20670828 PMC2957363

[B78] Nash Y. Schmukler E. Trudler D. Pinkas-Kramarski R. Frenkel D. (2017). DJ-1 deficiency impairs autophagy and reduces alpha-synuclein phagocytosis by microglia. *J. Neurochem.* 143 584–594. 10.1111/jnc.14222 28921554

[B79] Nayak D. Roth T. L. McGavern D. B. (2014). Microglia development and function. *Annu. Rev. Immunol.* 32 367–402. 10.1146/annurev-immunol-032713-120240 24471431 PMC5001846

[B80] O’Brien C. E. Wyss-Coray T. (2014). Sorting through the roles of beclin 1 in microglia and neurodegeneration. *J. Neuroimmune Pharmacol.* 9 285–292. 10.1007/s11481-013-9519-8 24385262 PMC4019692

[B81] Ojha C. R. Lapierre J. Rodriguez M. Dever S. M. Zadeh M. A. DeMarino C. (2017). Interplay between autophagy, exosomes and HIV-1 associated neurological disorders: new insights for diagnosis and therapeutic applications. *Viruses Basel* 9:176. 10.3390/v9070176 28684681 PMC5537668

[B82] Olanrewaju A. A. Hakami R. M. (2020). The messenger apps of the cell: extracellular vesicles as regulatory messengers of microglial function in the CNS. *J. Neuroimmune Pharmacol.* 15 473–486. 10.1007/s11481-020-09916-9 32337651 PMC7483331

[B83] Paolicelli R. C. Bolasco G. Pagani F. Maggi L. Scianni M. Panzanelli P. (2011). Synaptic pruning by microglia is necessary for normal brain development. *Science* 333 1456–1458. 10.1126/science.1202529 21778362

[B84] Park S. H. Lee Y. S. Yang H. J. Song G. J. (2021). Fluoxetine potentiates phagocytosis and autophagy in microglia. *Front. Pharmacol.* 12:770610. 10.3389/fphar.2021.770610 34899324 PMC8662994

[B85] Perluigi M. Di Domenico F. Butterfield D. A. (2015). mTOR signaling in aging and neurodegeneration: at the crossroad between metabolism dysfunction and impairment of autophagy. *Neurobiol. Dis.* 84 39–49. 10.1016/j.nbd.2015.03.014 25796566

[B86] Plaza-Zabala A. Sierra-Torre V. Sierra A. (2017). Autophagy and microglia: novel partners in neurodegeneration and aging. *Int. J. Mol. Sci.* 18:598. 10.3390/ijms18030598 28282924 PMC5372614

[B87] Pomilio C. Gorojod R. M. Riudavets M. Vinuesa A. Presa J. Gregosa A. (2020). Microglial autophagy is impaired by prolonged exposure to beta-amyloid peptides: evidence from experimental models and Alzheimer’s disease patients. *Geroscience* 42 613–632. 10.1007/s11357-020-00161-9 31975051 PMC7206478

[B88] Qin C. Liu Q. Hu Z. W. Zhou L. Q. Shang K. Bosco D. B. (2018). Microglial TLR4-dependent autophagy induces ischemic white matter damage via STAT1/6 pathway. *Theranostics* 8 5434–5451. 10.7150/thno.27882 30555556 PMC6276098

[B89] Ransohoff R. M. (2016). A polarizing question: do M1 and M2 microglia exist? *Nat. Neurosci.* 19 987–991. 10.1038/nn.4338 27459405

[B90] Rizzi L. Rosset I. Roriz-Cruz M. (2014). Global epidemiology of dementia: Alzheimer’s and vascular types. *Biomed. Res. Int.* 2014:908915. 10.1155/2014/908915 25089278 PMC4095986

[B91] Robbins N. M. Swanson R. A. (2014). Opposing effects of glucose on stroke and reperfusion injury: acidosis, oxidative stress, and energy metabolism. *Stroke* 45 1881–1886. 10.1161/STROKEAHA.114.004889 24743441 PMC4102697

[B92] Rubinsztein D. C. (2006). The roles of intracellular protein-degradation pathways in neurodegeneration. *Nature* 443 780–786. 10.1038/nature05291 17051204

[B93] Sanjuan M. A. Dillon C. P. Tait S. W. G. Moshiach S. Dorsey F. Connell S. (2007). Toll-like receptor signalling in macrophages links the autophagy pathway to phagocytosis. *Nature* 450 1253–1257. 18097414 10.1038/nature06421

[B94] Schafer D. P. Lehrman E. K. Kautzman A. G. Koyama R. Mardinly A. R. Yamasaki R. (2012). Microglia sculpt postnatal neural circuits in an activity and complement-dependent manner. *Neuron* 74 691–705. 10.1016/j.neuron.2012.03.026 22632727 PMC3528177

[B95] Shang J. Yamashita T. Zhai Y. Nakano Y. Morihara R. Li X. (2019). Acceleration of NLRP3 inflammasome by chronic cerebral hypoperfusion in Alzheimer’s disease model mouse. *Neurosci. Res.* 143 61–70. 10.1016/j.neures.2018.06.002 29885344

[B96] Shi H. Wang J. Wang J. Huang Z. Yang Z. (2018). IL-17A induces autophagy and promotes microglial neuroinflammation through ATG5 and ATG7 in intracerebral hemorrhage. *J. Neuroimmunol.* 323 143–151. 10.1016/j.jneuroim.2017.07.015 28778418

[B97] Shibutani S. T. Saitoh T. Nowag H. Muenz C. Yoshimori T. (2015). Autophagy and autophagy-related proteins in the immune system. *Nat. Immunol.* 16 1014–1024. 10.1038/ni.3273 26382870

[B98] Shimobayashi M. Hall M. N. (2014). Making new contacts: the mTOR network in metabolism and signalling crosstalk. *Nat. Rev. Mol. Cell Biol.* 15 155–162. 10.1038/nrm3757 24556838

[B99] Sierra A. Abiega O. Shahraz A. Neumann H. (2013). Janus-faced microglia: beneficial and detrimental consequences of microglial phagocytosis. *Front. Cell. Neurosci.* 7:6. 10.3389/fncel.2013.00006 23386811 PMC3558702

[B100] Sierra A. Encinas J. M. Deudero J. J. Chancey J. H. Enikolopov G. Overstreet-Wadiche L. S. (2010). Microglia shape adult hippocampal neurogenesis through apoptosis-coupled phagocytosis. *Cell Stem Cell* 7 483–495. 10.1016/j.stem.2010.08.014 20887954 PMC4008496

[B101] Sikkema A. H. Stoffels J. M. J. Wang P. Basedow F. J. Bulsink R. Bajramovic J. J. (2018). Fibronectin aggregates promote features of a classically and alternatively activated phenotype in macrophages. *J. Neuroinflamm.* 15:218. 10.1186/s12974-018-1238-x 30071854 PMC6091019

[B102] Song S. Yu L. Hasan M. N. Paruchuri S. S. Mullett S. J. Sullivan M. L. G. (2022). Elevated microglial oxidative phosphorylation and phagocytosis stimulate post-stroke brain remodeling and cognitive function recovery in mice. *Commun. Biol.* 5:35. 10.1038/s42003-021-02984-4 35017668 PMC8752825

[B103] Song W. M. Colonna M. (2018). The identity and function of microglia in neurodegeneration. *Nat. Immunol.* 19 1048–1058. 10.1038/s41590-018-0212-1 30250185

[B104] Su P. Zhang J. Wang D. Zhao F. Cao Z. Aschner M. (2016). The role of autophagy in modulation of neuroinflammation in microglia. *Neuroscience* 319 155–167. 10.1016/j.neuroscience.2016.01.035 26827945

[B105] Su S. H. Wu Y. F. Lin Q. Wang D. P. Hai J. (2019). URB597 protects against NLRP3 inflammasome activation by inhibiting autophagy dysfunction in a rat model of chronic cerebral hypoperfusion. *J. Neuroinflamm.* 16:260. 10.1186/s12974-019-1668-0 31815636 PMC6900848

[B106] Switon K. Kotulska K. Janusz-Kaminska A. Zmorzynska J. Jaworski J. (2017). Molecular neurobiology of mTOR. *Neuroscience* 341 112–153. 10.1016/j.neuroscience.2016.11.017 27889578

[B107] Tang Y. Liu J. Wang Y. Yang L. Han B. Zhang Y. (2021). PARP14 inhibits microglial activation via LPAR5 to promote post-stroke functional recovery. *Autophagy* 17 2905–2922. 10.1080/15548627.2020.1847799 33317392 PMC8525999

[B108] Thammisetty S. S. Renaud L. Picher-Martel V. Weng Y. C. Calon F. Saikali S. (2021). Targeting TDP-43 pathology alleviates cognitive and motor deficits caused by chronic cerebral hypoperfusion. *Neurotherapeutics* 18 1095–1112. 10.1007/s13311-021-01015-8 33786804 PMC8423945

[B109] Tian D. S. Li C. Y. Qin C. Murugan M. Wu L. J. Liu J. L. (2016). Deficiency in the voltage-gated proton channel Hv1 increases M2 polarization of microglia and attenuates brain damage from photothrombotic ischemic stroke. *J. Neurochem.* 139, 96–105. 10.1111/jnc.13751 27470181 PMC5037018

[B110] Ulland T. K. Song W. M. Huang S. C. Ulrich J. D. Sergushichev A. Beatty W. L. (2017). TREM2 maintains microglial metabolic fitness in Alzheimer’s disease. *Cell* 170 649–663. 10.1016/j.cell.2017.07.023 28802038 PMC5573224

[B111] Urabe T. (2012). Molecular mechanism and new protective strategy for ischemic white matter damages. *Rinsho Shinkeigaku* 52 908–910. 10.5692/clinicalneurol.52.908 23196463

[B112] Viscomi M. T. D’Amelio M. Cavallucci V. Latini L. Bisicchia E. Nazio F. (2012). Stimulation of autophagy by rapamycin protects neurons from remote degeneration after acute focal brain damage. *Autophagy* 8 222–235. 10.4161/auto.8.2.18599 22248716

[B113] Vlassov A. V. Magdaleno S. Setterquist R. Conrad R. (2012). Exosomes: current knowledge of their composition, biological functions, and diagnostic and therapeutic potentials. *Biochim. Biophys. Acta* 1820 940–948. 10.1016/j.bbagen.2012.03.017 22503788

[B114] Walsh J. G. Muruve D. A. Power C. (2014). Inflammasomes in the CNS. *Nat. Rev. Neurosci.* 15 84–97. 10.1038/nrn3638 24399084

[B115] Wang H. Liu Q. Zhang X. (2020). C1q/tumor necrosis factor-related protein-1 attenuates microglia autophagy and inflammatory response by regulating the Akt/mTOR pathway. *Life Sci.* 256:117992. 10.1016/j.lfs.2020.117992 32569781

[B116] Wang J. (2010). Preclinical and clinical research on inflammation after intracerebral hemorrhage. *Prog. Neurobiol.* 92 463–477. 10.1016/j.pneurobio.2010.08.001 20713126 PMC2991407

[B117] Wang N. He J. Pan C. Wang J. Ma M. Shi X. (2019). Resveratrol activates autophagy via the AKT/mTOR signaling pathway to improve cognitive dysfunction in rats with chronic cerebral hypoperfusion. *Front. Neurosci.* 13:859. 10.3389/fnins.2019.00859 31481868 PMC6710371

[B118] Wang S. Wang C. Wang L. Cai Z. (2020). Minocycline inhibits mTOR signaling activation and alleviates behavioral deficits in the wistar rats with acute ischemia stroke. *CNS Neurol. Disord. Drug Targets* 19 791–799. 10.2174/1871527319999200831153748 32867663

[B119] Wang X. X. Zhang B. Xia R. Jia Q. Y. (2020). Inflammation, apoptosis and autophagy as critical players in vascular dementia. *Eur. Rev. Med. Pharmacol. Sci.* 24 9601–9614. 10.26355/eurrev_202009_23048 33015803

[B120] Wang Z. Yuan B. Fu F. Huang S. Yang Z. (2017). Hemoglobin enhances miRNA-144 expression and autophagic activation mediated inflammation of microglia via mTOR pathway. *Sci. Rep.* 7:11861. 10.1038/s41598-017-12067-2 28928406 PMC5605685

[B121] West P. K. McCorkindale A. N. Guennewig B. Ashhurst T. M. Viengkhou B. Hayashida E. (2022). The cytokines interleukin-6 and interferon-alpha induce distinct microglia phenotypes. *J. Neuroinflammation* 19:96. 10.1186/s12974-022-02441-x 35429976 PMC9013466

[B122] Xia C. Y. Zhang S. Chu S. F. Wang Z. Z. Song X. Y. Zuo W. (2016). Autophagic flux regulates microglial phenotype according to the time of oxygen-glucose deprivation/reperfusion. *Int. Immunopharmacol.* 39 140–148. 10.1016/j.intimp.2016.06.030 27474951

[B123] Xiang J. Liu X. Ren J. Chen K. Wang H.-L. Miao Y.-Y. (2019). How does estrogen work on autophagy? *Autophagy* 15 197–211. 10.1080/15548627.2018.1520549 30208759 PMC6333457

[B124] Xie L. Sun F. Wang J. Mao X. Xie L. Yang S. H. (2014). mTOR signaling inhibition modulates macrophage/microglia-mediated neuroinflammation and secondary injury via regulatory T cells after focal ischemia. *J. Immunol.* 192 6009–6019. 10.4049/jimmunol.1303492 24829408 PMC4128178

[B125] Xin D. Chu X. Bai X. Ma W. Yuan H. Qiu J. (2018). l-Cysteine suppresses hypoxia-ischemia injury in neonatal mice by reducing glial activation, promoting autophagic flux and mediating synaptic modification via HS formation. *Brain Behav. Immun.* 73 222–234. 10.1016/j.bbi.2018.05.007 29751053

[B126] Xu J. Camfield R. Gorski S. M. (2018). The interplay between exosomes and autophagy - partners in crime. *J. Cell Sci.* 131:jcs215210. 10.1242/jcs.215210 30076239

[B127] Xu Y. Propson N. E. Du S. Xiong W. Zheng H. (2021). Autophagy deficiency modulates microglial lipid homeostasis and aggravates tau pathology and spreading. *Proc. Natl. Acad. Sci. U.S.A.* 118:e2023418118. 10.1073/pnas.2023418118 34187889 PMC8271658

[B128] Yan W. Fan J. Zhang X. Song H. Wan R. Wang W. (2021). Decreased neuronal synaptosome associated protein 29 contributes to poststroke cognitive impairment by disrupting presynaptic maintenance. *Theranostics* 11 4616–4636. 10.7150/thno.54210 33754017 PMC7978312

[B129] Yan W. Zhang H. Bai X. Lu Y. Dong H. Xiong L. (2011). Autophagy activation is involved in neuroprotection induced by hyperbaric oxygen preconditioning against focal cerebral ischemia in rats. *Brain Res.* 1402 109–121. 10.1016/j.brainres.2011.05.049 21684529

[B130] Yang G. Wang N. Seto S. W. Chang D. Liang H. (2018). Hydroxysafflor yellow a protects brain microvascular endothelial cells against oxygen glucose deprivation/reoxygenation injury: involvement of inhibiting autophagy via class I PI3K/Akt/mTOR signaling pathway. *Brain Res. Bull.* 140 243–257. 10.1016/j.brainresbull.2018.05.011 29775658

[B131] Yang S. Qin C. Hu Z. W. Zhou L. Q. Yu H. H. Chen M. (2021). Microglia reprogram metabolic profiles for phenotype and function changes in central nervous system. *Neurobiol. Dis.* 152;105290. 10.1016/j.nbd.2021.105290 33556540

[B132] Yang Z. Klionsky D. J. (2010). Mammalian autophagy: core molecular machinery and signaling regulation. *Curr. Opin. Cell Biol.* 22 124–131. 10.1016/j.ceb.2009.11.014 20034776 PMC2854249

[B133] Yang Z. Liu B. Zhong L. Shen H. Lin C. Lin L. (2015a). Toll-like receptor-4-mediated autophagy contributes to microglial activation and inflammatory injury in mouse models of intracerebral haemorrhage. *Neuropathol. Appl. Neurobiol.* 41 e95–e106. 10.1111/nan.12177 25185720

[B134] Yang Z. Zhong L. N. Zhong S. C. Xian R. H. Yuan B. Q. (2015b). Hypoxia induces microglia autophagy and neural inflammation injury in focal cerebral ischemia model. *Exp. Mol. Pathol.* 98 219–224. 10.1016/j.yexmp.2015.02.003 25666359

[B135] Yang Z. Zhang N. Shen H. Lin C. Lin L. Yuan B. (2014a). Microglial activation with reduction in autophagy limits white matter lesions and improves cognitive defects during cerebral hypoperfusion. *Curr. Neurovasc. Res.* 11 223–229. 10.2174/1567202611666140520124407 24845855

[B136] Yang Z. Zhao T. Zou Y. Zhang J. H. Feng H. (2014b). Curcumin inhibits microglia inflammation and confers neuroprotection in intracerebral hemorrhage. *Immunol. Lett.* 160 89–95. 10.1016/j.imlet.2014.03.005 24680995

[B137] Yang Z. Zhao T. Z. Zou Y. J. Zhang J. H. Feng H. (2014c). Hypoxia induces autophagic cell death through hypoxia-inducible factor 1alpha in microglia. *PLoS One* 9:e96509. 10.1371/journal.pone.0096509 24818601 PMC4018331

[B138] Yuan B. Shen H. Lin L. Su T. Zhong L. Yang Z. (2017). Autophagy promotes microglia activation through Beclin-1-Atg5 pathway in intracerebral hemorrhage. *Mol. Neurobiol.* 54 115–124. 10.1007/s12035-015-9642-z 26732594

[B139] Zang J. Wu Y. Su X. Zhang T. Tang X. Ma D. (2020). Inhibition of PDE1-B by vinpocetine regulates microglial exosomes and polarization through enhancing autophagic flux for neuroprotection against ischemic stroke. *Front. Cell Dev. Biol.* 8:616590. 10.3389/fcell.2020.616590 33614626 PMC7889976

[B140] Zang L. Wang J. Ren Y. Liu W. Yu Y. Zhao S. (2019). Activated toll-like receptor 4 is involved in oridonin-induced phagocytosis via promotion of migration and autophagy-lysosome pathway in RAW264.7 macrophages. *Int. Immunopharmacol.* 66 99–108. 10.1016/j.intimp.2018.11.014 30445312

[B141] Zeng Y. Zhang W. Xue T. Zhang D. Lv M. Jiang Y. (2022). Sphk1-induced autophagy in microglia promotes neuronal injury following cerebral ischaemia-reperfusion. *Eur. J. Neurosci.* 56 4287–4303. 10.1111/ejn.15749 35766986

[B142] Zhang S. Q. Ding F. F. Liu Q. Tian Y. Y. Wang W. Qin C. (2019). Autophagy inhibition exerts neuroprotection on white matter ischemic damage after chronic cerebral hypoperfusion in mice. *Brain Res.* 1721:146337. 10.1016/j.brainres.2019.146337 31319064

[B143] Zhang Y. Bi J. Huang J. Tang Y. Du S. Li P. (2020). Exosome: a review of its classification, isolation techniques, storage, diagnostic and targeted therapy applications. *Int. J. Nanomedicine* 15 6917–6934. 10.2147/IJN.S264498 33061359 PMC7519827

[B144] Zhang Y. Shen K. Bai Y. Lv X. Huang R. Zhang W. (2016). Mir143-BBC3 cascade reduces microglial survival via interplay between apoptosis and autophagy: implications for methamphetamine-mediated neurotoxicity. *Autophagy* 12 1538–1559. 10.1080/15548627.2016.1191723 27464000 PMC5082785

[B145] Zhao Y. Yang G. (2021). Potential of extracellular vesicles in the Parkinson’s disease - Pathological mediators and biomarkers. *Neurochem. Int.* 144:104974. 10.1016/j.neuint.2021.104974 33485881

[B146] Zhao Y. Zhang J. Zheng Y. Zhang Y. Zhang X. J. Wang H. (2021). NAD^+^ improves cognitive function and reduces neuroinflammation by ameliorating mitochondrial damage and decreasing ROS production in chronic cerebral hypoperfusion models through Sirt1/PGC-1a pathway. *J. Neuroinflammation* 18:207. 10.1186/s12974-021-02250-8 34530866 PMC8444613

[B147] Zheng H. Cheng B. Li Y. Li X. Chen X. Zhang Y. W. (2018). TREM2 in Alzheimer’s disease: microglial survival and energy metabolism. *Front. Aging Neurosci.* 10:395. 10.3389/fnagi.2018.00395 30532704 PMC6265312

[B148] Zheng L. Jia J. Chen Y. Liu R. Cao R. Duan M. (2022). Pentoxifylline alleviates ischemic white matter injury through up-regulating Mertk-mediated myelin clearance. *J. Neuroinflamm.* 19:128. 10.1186/s12974-022-02480-4 35642056 PMC9153105

[B149] Zhou T. Lin D. Chen Y. Peng S. Jing X. Lei M. (2019). alpha-synuclein accumulation in SH-SY5Y cell impairs autophagy in microglia by exosomes overloading miR-19a-3p. *Epigenomics* 11 1661–1677. 10.2217/epi-2019-0222 31646884

[B150] Zhou X. Zhou J. Li X. Guo C. A. Fang T. Chen Z. (2011). GSK-3β inhibitors suppressed neuroinflammation in rat cortex by activating autophagy in ischemic brain injury. *Biochem. Biophys. Res. Commun.* 411 271–275. 10.1016/j.bbrc.2011.06.117 21723251

[B151] Zhou X. Y. Luo Y. Zhu Y. M. Liu Z. H. Kent T. A. Rong J. G. (2017). Inhibition of autophagy blocks cathepsins-tBid-mitochondrial apoptotic signaling pathway via stabilization of lysosomal membrane in ischemic astrocytes. *Cell Death Dis.* 8:e2618. 10.1038/cddis.2017.34 28206988 PMC5386481

[B152] Zhou Y. Ulland T. K. Colonna M. (2018). TREM2-dependent effects on microglia in Alzheimer’s disease. *Front. Aging Neurosci.* 10:202. 10.3389/fnagi.2018.00202 30038567 PMC6046445

[B153] Zhu Y. Yu J. Gong J. Shen J. Ye D. Cheng D. (2021). PTP1B inhibitor alleviates deleterious microglial activation and neuronal injury after ischemic stroke by modulating the ER stress-autophagy axis via PERK signaling in microglia. *Aging* 13 3405–3427. 10.18632/aging.202272 33495405 PMC7906217

[B154] Zlokovic B. V. (2008). The blood-brain barrier in health and chronic neurodegenerative disorders. *Neuron* 57 178–201. 10.1016/j.neuron.2008.01.003 18215617

[B155] Zrzavy T. Hametner S. Wimmer I. Butovsky O. Weiner H. L. Lassmann H. (2017). Loss of ‘homeostatic’ microglia and patterns of their activation in active multiple sclerosis. *Brain* 140 1900–1913. 10.1093/brain/awx113 28541408 PMC6057548

